# Alpha-Defensin 5 Expression is Regulated by microRNAs in the Caco-2 Intestinal Epithelial Cell Line

**Published:** 2016-04

**Authors:** Donald R B Miles, Jun Shen, Alice Y. Chuang, Fenshi Dong, Feng Wu, John Kwon

**Affiliations:** 1 Johns Hopkins School of Medicine, USA; 2 University of Chicago, USA; 3 University of Texas, Southwestern Medical Center, USA

**Keywords:** Defensin, Inflammatory bowel disease, MicroRNA regulation

## Abstract

**Background:**

In inflammatory bowel disease (IBD), an inappropriate immune response leads to chronic mucosal inflammation. This response may be partly due to dysregulation of defensins, which are endogenously produced antimicrobial peptides. This study determined whether microRNAs (miRNAs) regulate α-defensin 5 (DEFA5), which could further implicate both in IBD pathogenesis.

**Methods:**

Induction of DEFA5 mRNA and protein expression was determined in Caco-2 cells. An *in silico* analysis identified putative miRNA binding sites of DEFA5. Expression of these miRNAs was assessed in Caco-2 cells. Regulation of DEFA5 expression by these miRNAs was measured by luciferase assays. Caco-2 cells were transfected with miR-124 and miR-924 mimics, and DEFA5 mRNA and protein expression was measured.

**Results:**

DEFA5 mRNA and protein expression was inducible in Caco-2 cells. Fifteen putative miRNA binding sites were found in DEFA5. The expression of miR-124 and miR-924 decreased following induction. Transfection of a luciferase construct containing the DEFA5 miRNA binding sites resulted in a decrease in luciferase activity compared to transfection of the empty vector. Transfection of a reporter construct containing mismatched miRNA binding sites resulted in restoration of luciferase activities. Transfection of miRNA mimics decreased DEFA5 mRNA expression and protein expression.

**Conclusions:**

miR-124 and miR-924 negatively regulate DEFA5 mRNA and protein expression. These data implicate miRNAs in intestinal innate immune regulation and IBD pathogenesis.

## Introduction

Crohn's disease (CD), one of the two types of inflammatory bowel disease (IBD), is characterized by inappropriate and continuous activation of the mucosal immune system [1,2]. In the normal small intestine, Paneth cells, an important part of the innate mucosal immune system, maintain microbial homeostasis by secreting defensins, a class of cationic peptides with a broad spectrum of antimicrobial activity [3]. The two broad categories of defensins include α and β-defensins. In the small intestine, α-defensin 5 (DEFA5) is secreted into the lumen of intestinal crypts by Paneth cells after activation of the NOD2 receptor by bacterial endotoxins [4-6]. The α-defensins are chemotactic for cells of both innate and adaptive immune systems, including macrophages and T cells [7]. They are involved in several processes that maintain intestinal homeostasis, including regulation of gut flora, intestinal inflammation, stem cell protection, and crypt development [8-10]. In genetically susceptible individuals, changes in intestinal bacterial flora, including commensal bacteria, play a role in IBD pathogenesis. These changes contribute to the initiation and perpetuation of chronic mucosal inflammation [11-14]. Since defensins maintain the balance between commensal microbes and intestinal mucosa, their dysregulation could contribute to IBD pathogenesis. Defensin expression has been shown to be altered in the setting of NOD2 mutations and changes in certain signaling pathways, but no research has demonstrated the role of microRNAs in defensin regulation. However, SNPs within the 3′ UTR of DEFA5, have been linked with increased susceptibility to IBD, suggesting the possible role of miRNA regulation [15]. Additionally, post-transcriptional regulation of gene expression, such as that by microRNAs, has been shown to play an important role in IBD pathogenesis [16-18]. Altered miRNA expression profiles exist in active IBD indicating that changes in miRNA expression are associated with disease [17-21]. Therefore, the purpose of this study is to examine the role of miRNAs in regulating DEFA5 expression using Caco-2 cells as a model of Paneth cells.

## Materials and Methods

### Cell lines and cell culture

Human colonic epithelial cell lines (Caco-2, HT29, and HCT116 cells) were previously obtained and used for this study. The Caco-2 cells were cultured in Dulbecco's Modified Eagle Medium (DMEM) (Cellgro, Manassas, VA), while the HT29 and HCT116 cells were cultured in McCoy's 5A Medium (Cellgro, Manassas, VA). The culture medium was supplemented with 10% fetal bovine serum (FBS) and 1% penicillin/streptomycin. All cell lines were cultured at 37°C in a humidified atmosphere containing 5% CO_2_ and the medium was changed every two days. FGF-2 (10 ng/ml, R&D Systems, Minneapolis, MN) was solubilized in culture medium containing heparin sodium salt (1.25 ug/ml; Sigma, St. Louis, MO). FGF-2 was added daily beginning at 24 hours post-plating and until cells were harvested for assay, as described previously [22]. Caco-2 cells have previously been used as a model of Paneth cells, and differentiation into a Paneth cell phenotype has been confirmed with Paneth cell markers, including expression of alpha-defensin 5, alpha-defensin 6, lysozyme, and sPLA2 in previous studies [22-24]. For experiments in which Caco-2 cells were stimulated with TNFα, Caco-2 cells were first treated with FGF-2 for 72 hours, followed by addition of medium alone (control) or medium containing TNFα (R&D Systems, Minneapolis, MN) at 50 ng/mL. Cells were harvested at four hours for mRNA and 8 hours for protein analyses. Prior to cell harvesting for Western blot, Caco-2 cells were treated with Brefeldin-A (Sigma) at 5 ug/ml for a total of 8 hours (replenished every four hours) to prevent secretion of DEFA5 into the cellular medium.

### Total RNA

Total RNA was isolated using TRIzol reagent (Life Technologies, Grand Island, NY) according to the manufacturer's protocol. RNA concentrations were determined using a Nano-Drop 1000 spectrophotometer (Thermo Fisher Scientific, Wilmington, DE). The isolated RNA was stored at -80°C until use in Quantitative Reverse Transcription-Polymerase Chain Reaction (qRT-PCR).

### Quantitative reverse transcription-polymerase chain reaction for mRNA

One microgram of total RNA was converted to complementary DNA (cDNA) using the qScript cDNA SuperMix (Quanta Biosciences, Gaithersburg, MD). qRT-PCR was performed using SYBR Green Power PCR Master Mix (Applied Biosystems, Foster City, CA). The qRT-PCR amplifications were performed on the LightCycler 480 realtime PCR system (Roche, Indianapolis, IN). The cycles passing threshold (Ct) were recorded and the expression of GAPDH was used as an internal control. The primers for DEFA5 were 5′-ACCCAGAAGCAGTCTGGGGAAGA-3′ (forward) and 5′ – GGTGGCTCTTGCCTGAGAACCTGA-3′ (reverse). The primers for GAPDH were 5′ – CGACCACTTTGTCAAGCTCA-3′ (forward) and 5′-AGGGGAGATTCAGTGTGGTG-3′ (reverse).

### Quantitative reverse transcription-polymerase chain reaction for miRNA

One microgram of total RNA was converted to cDNA using the NCode VILO miRNA cDNA Synthesis Kit (Life Technologies)followed by qPCR using the NCode EXPRESS SYBR GreenER miRNA qRT-PCR kit (Life Technologies). The cycles passing threshold were recorded and the expression of miRNAs was calculated relative to U6B, a ubiquitously expressed small nuclear RNA. The forward primer for miR-124 was 5′- TAAGGCACGCGGTGAATGCC-3′. The forward primer for miR-924 was 5′-CCTCTGCCCTCTAAAGGTTTGC-3′. The forward primer for U6B was 5′-CGCAAGGATGACACGCAAATTCG-3′. The reverse primer was the NCode miRNA universal qPCR primer (Invitrogen). Data are presented as target miRNA or mRNA expression=2ΔCt, with ΔCt=(U6B or GAPDH Ct – target miRNA or mRNA Ct). qRT-PCR was carried out in triplicate for each sample for both the U6B or GAPDH control and each miRNA or mRNA.

### Western blot

Cells were lysed in cold RIPA buffer (Thermo Fisher Scientific, Rockford, IL) supplemented with 1% protease and phosphatase inhibitor cocktail (Thermo Fisher Scientific). Protein concentrations were determined with the BCA protein assay (Thermo Fisher Scientific). Cell lysates were suspended in × Laemmli sample buffer (Bio-Rad, Hercules, CA) containing 5% 2-mercaptoethanol and boiled for 5 minutes. After heat denaturation, total protein lysates (30 ug/lane) were subjected to tricine-SDS-PAGE [25]. The proteins were then transferred electrophoretically to 0.2 um PVDF membranes. Membranes were blocked in blocking buffer (LI-COR Biosciences, Lincoln, NE) diluted 1:1 in × PBS for 1 hour at room temperature. The blots were incubated with mouse anti-DEFA5 (Santa Cruz Biotechnologies, Santa Cruz, CA; 1:200 dilution), goat anti-actin (Sigma; 1:1000 dilution), and mouse anti-GAPDH (Life Technologies; 1:1000 dilution) overnight at 4°C. After washing with PBST, blots were incubated with Alexa Fluor 680 (Life Technologies) and IRDye 800CW (LI-COR Biosciences) conjugated secondary antibodies (1:10,000 dilution) for 1 hour at room temperature. The band intensities were quantified using an Odyssey infrared imaging system (LI-COR Biosciences) and analyzed using Image Studio Lite 3.1 (LI-COR Biosciences).

### DEFA5 3′ UTR construct

The full-length 3′ UTR of DEFA5 (nucleotides 328-451 of NM_021010) was cloned into the PmeI and SacI sites downstream of the dual firefly and Renilla luciferase reporter vector, pmirGLO construct (Promega, Madison, WI) by GenScript. The full length 3′ UTR was also used as a template to mutate the entire seed region of the putative miR-124 and miR-924 binding sites.

### miRNA mimic and luciferase construct transfection

Synthesized RNA duplexes of miRNA mimics (agomiRs) to miR-124 and miR-924 and the negative control were obtained from Sigma (St. Louis, MO). A miRNA mimic or a luciferase construct was transfected into Caco-2 cells using Lipofectamine 2000 reagent (Life Technologies) according to the manufacturer's guidelines. At 24 hours post-transfection, cells were harvested for RNA and protein analyses or were harvested for measurement of luciferase activities.

### Luciferase reporter assay

Caco-2 cells were lysed in passive lysis buffer and then analyzed for the firefly and Renilla luciferase activities using the Dual-Luciferase Reporter Assay System (Promega) on the GloMax-Multi Detection System Luminometer (Promega) according to the manufacturer's instructions. The firefly luciferase activity was normalized to the renilla luciferase activity.

### Statistical analysis

All experiments were performed with four biological repeats in triplicate. R (R Development Core Team, Vienna, Austria) was used for statistical analysis [26]. Statistical significance was determined by 2-tailed Student's t tests (for comparison of two conditions) and one-way ANOVA for comparison of multiple groups. Data are presented as mean ± standard error of the mean. P< 0.05 was considered significant.

## Results

### DEFA5 expression in colonic epithelial cells

**Figure 1:**
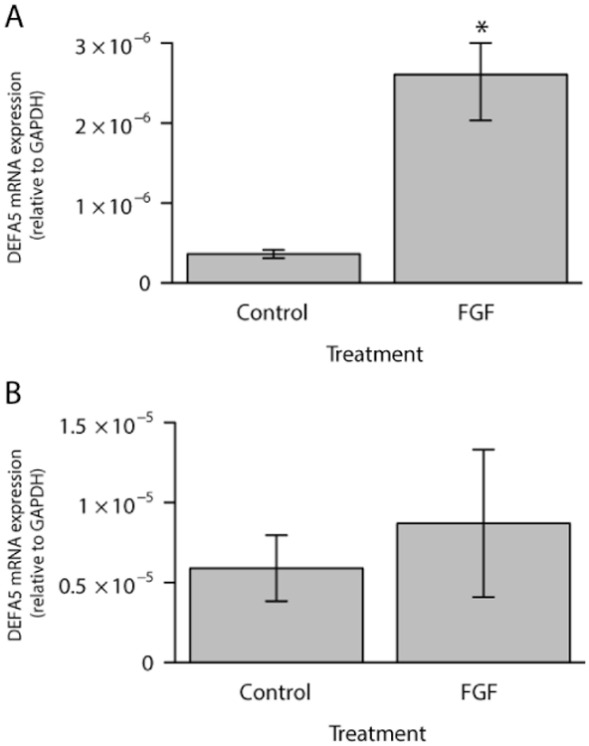
Endogenous DEFA5 expression (control) and following 72 hours treatment with FGF-2 (FGF) in Caco-2 (A) and HCT116 (B) cell lines. Results are mean ± standard error, n=4. *P<0.05

**Figure 2:**
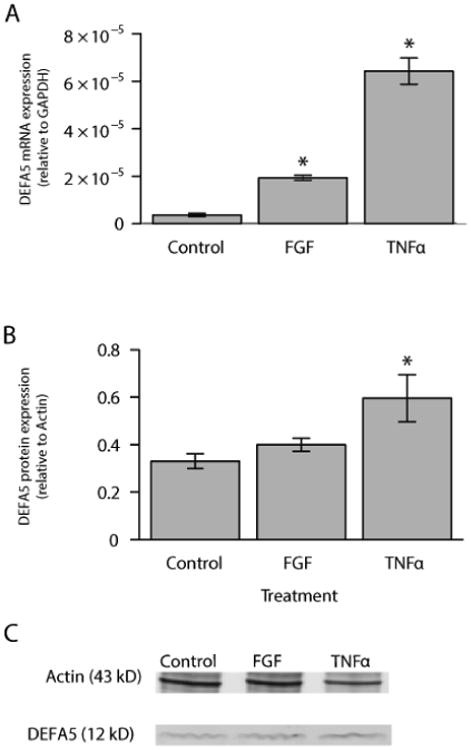
DEFA5 mRNA and protein expression in Control, FGF-2, and FGF-2/TNFα-stimulated Caco-2 colonic epithelial cells. (A) The mRNA expression of DEFA5 was assessed at 72 hours after FGF-2 stimulation or at 72 hours of FGF-2 plus 4 hours of TNFα stimulation. (B) Densitometric analysis of protein expression following induction of Caco-2 cells presented in graphical form. (C) Assessment of DEFA5 protein expression by Western blot. Actin served as a loading control. Results are mean ± standard error, n=4, *P<0.05.

Alpha defensin expression and inducible expression by FGF-2 were ascertained in three different colonic epithelial cell lines: Caco-2, HCT116, and HT29. Both the Caco-2 and HCT116 cell lines expressed DEFA5, while the HT29 cell lines did not express DEFA5. DEFA5 expression was significantly increased in Caco-2 cells following treatment for 72 hours with FGF-2, as previously demonstrated by Brodrick et al. and Tan et al. (Figure 1) [22-24]. Because Caco-2 cells expressed the α-defensins at a highly inducible level, all subsequent work was conducted in that cell line. Next, we confirmed that DEFA5 mRNA expression was inducible in Caco-2 cells by both FGF-2 and TNFα. Specifically, DEFA5 mRNA expression after treatment with FGF-2 (10 ng/ml) for 72 hours and FGF-2 for 72 hours plus TNFα (50 ng/ml) for 4 hours was measured (Figure 2A). Caco-2 cells stimulated with FGF-2 resulted in a statistically significant 5.3 fold increase and those stimulated with FGF-2 followed by TNFα resulted in a statistically significant 17.5 fold increase (Figure 2A). Treatment of Caco-2 cells with FGF-2 in combination with TNFα for 8 hours resulted in a statistically significant 1.8 fold increase in protein expression as measured by densitometry performed on Western blots (Figure 2B). An example Western blot of Actin and DEFA5 is shown (Figure 2C).

### Expressions of miRNAs that bind to DEFA5 3′ UTR in Caco-2 cells

**Figure 3:**
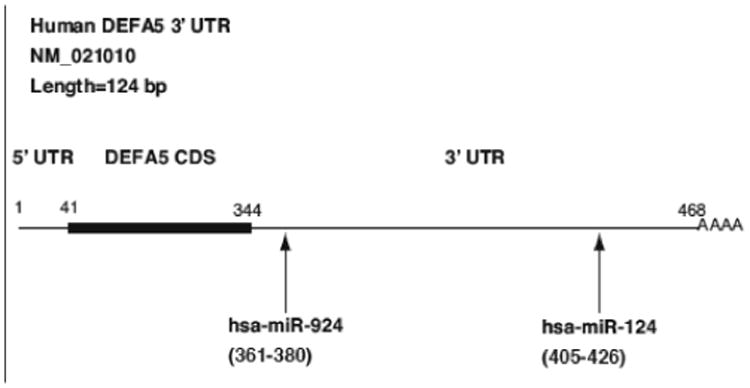
Schematic representation of DEFA5 mRNA with putative miRNA binding sites in 3′ UTR

**Figure 4:**
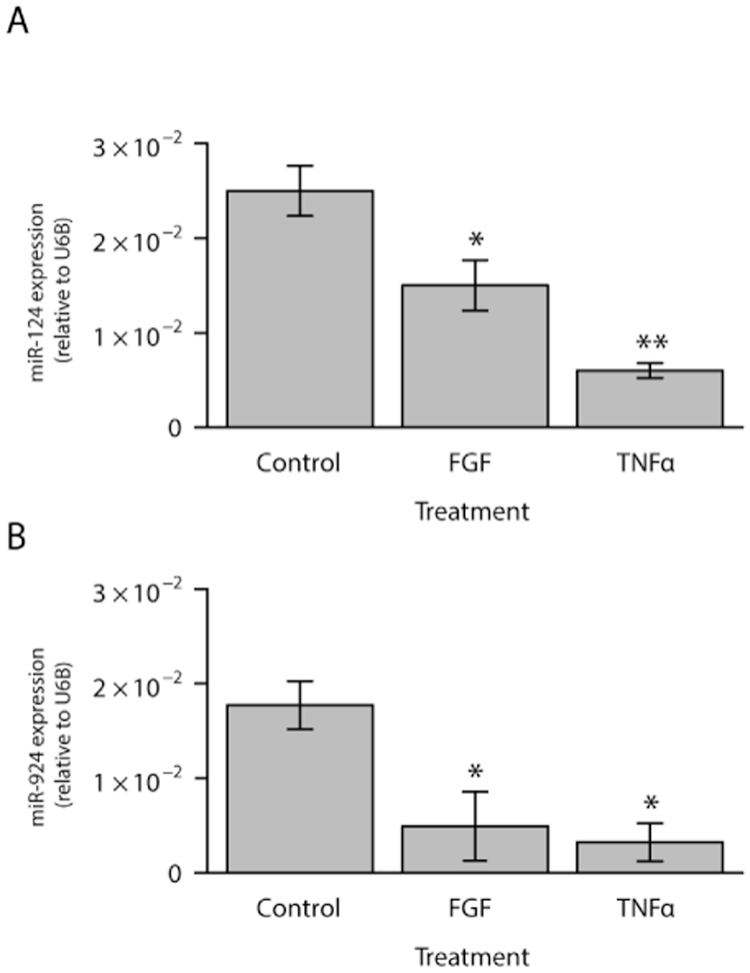
DEFA5-3′-UTR-associated miRNA expression in stimulated Caco-2 colonic epithelial cells. The expression of DEFA5-associated miRNAs was assessed at 72 hours after FGF-2 stimulation or at 72 hours of FGF-2 plus 4 hours of TNFα stimulation. (A) demonstrates the expression of miR-124 and (B) indicates the expression of miR-924. Results are mean ± standard error, n=4, *P<0.05, **P<0.01

**Figure 5:**
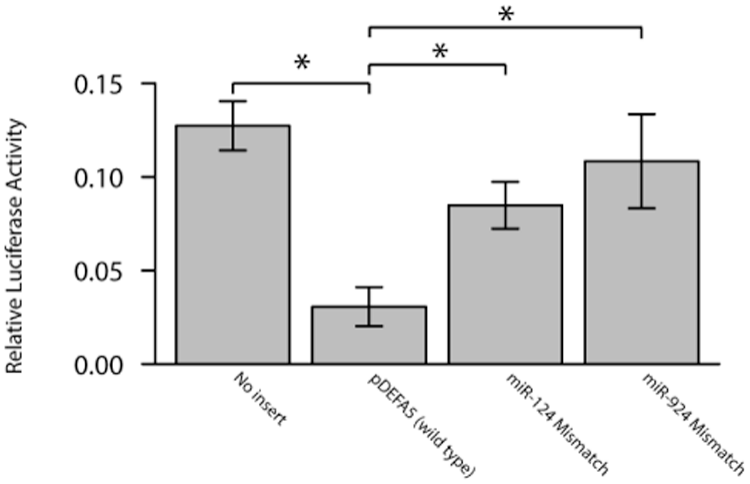
Luciferase reporter activity in the pMIR-DEFA5-3′ UTR reporter construct. Luciferase activity (normalized to Renilla luciferase activity) data is presented relative to the pMIR Reporter. Results are mean ± standard error, n=4, *P<0.05.

**Figure 6:**
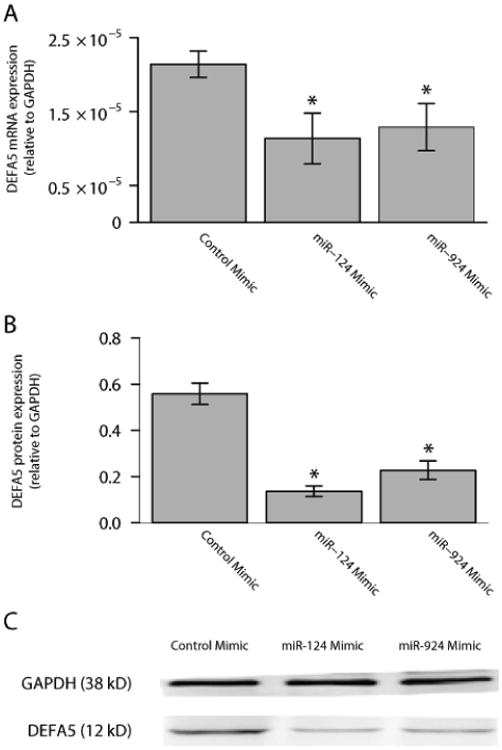
miR-124 and miR-924 inhibition of DEFA5 mRNA and protein expression. (A) DEFA5 mRNA expression was significantly reduced in Caco-2 cells transfected with either miR-124 or miR-924 mimics. * P<0.05 (B) DEFA5 protein expression was significantly reduced in Caco-2 cells transfected with either miR-124 or miR-924 mimics. (C) Assessment of DEFA5 protein expression by Western blot. GAPDH served as a loading control. Results are mean ± standard error, n=4, *P<0.05.

An in silico analysis utilizing miRBase and TargetScan identified fifteen putative miRNA binding sites in the 3′ UTR of DEFA5. We identified the miRNAs with putative binding sites in the DEFA5 3′UTR and searched for miRNAs that were down-regulated in response to FGF-2 and TNFα. Of those miRNAs, we identified miR-124 and mi-924 (Figure 3) [27-30]. The other thirteen miRNAs were not examined further due to low relative expression. Because we hypothesized that the FGF- and TNFα- induced increase in DEFA5 expression may be influenced by a corresponding down-regulation of regulatory miRNAs, we examined the expression of miR-124 and miR-924 in response to FGF-2 and TNFα (Figure 4). DEFA5 induction was accompanied by a significant decrease in expression of miR-124 and miR-924 compared to unstimulated cells (Figures 4A and 4B). Our data demonstrate an inverse correlation between DEFA5 expression and the expression of miR-124 and miR-924, suggesting that DEFA5 may be regulated by miRNAs. Additionally, these data raise the possibility that the effect of FGF-2 and TNFα may be at least in part mediated by a down-regulation of DEFA5-associated miRNAs. To determine the effect of endogenous miRNAs on DEFA5 expression, a luciferase reporter construct containing the wild-type DEFA5 3′UTR was transfected into unstimulated Caco-2 cells. Transfecting this construct resulted in a 76% decrease in relative luciferase activity compared to transfection of the empty vector, which contained no 3′ UTR (Figure 5). These data indicate that the DEFA5 3′ UTR has functional miRNA binding sites to which endogenous miRNAs can bind and negatively regulate DEFA5 gene expression. Next, a luciferase reporter construct containing the DEFA5 3′ UTR with mutated binding sites of either miR-124 or miR-924 were transfected into Caco-2 cells. Transfection of the mismatched miR-124 construct resulted in a restoration to 66% of the relative luciferase activities of the empty vector (Figure 5). Transfection of the mismatched miR-924 construct restored relative luciferase activities to 85% of the empty vector (Figure 5). These data indicate that mutation of the binding sites led to a loss of regulation by each miRNA. This increase in relative luciferase activity compared to transfection of the empty vector was statistically significant in both miRNAs tested. We next tested whether miR-124 and miR-924 can negatively regulate basal DEFA5 expression. A miR-124 or miR-924 mimic was transiently transfected into Caco-2 cells and DEFA5 mRNA expression assessed (Figure 6). The miR-124 and miR-924 mimics significantly decreased DEFA5 mRNA expression by 47% and 50%, respectively. In addition, transfection of the miR-124 mimic resulted in a 75% decrease in DEFA5 protein expression as demonstrated by densitometry and transfection of the miR-924 mimic resulted in a 60% decrease in DEFA5 protein expression. Overall, these data suggest that miRNAs negatively regulate the mRNA and protein expression of DEFA5.

## Discussion

To the best of our knowledge, this is the first study to show miRNA regulation of DEFA5. We hypothesized that miRNAs regulate DEFA5 expression. We compared the expression of DEFA5 and miRNAs with putative binding sites in the 3′ UTR of DEFA5 in Caco-2 cells. We found that the expression of miR-124 and miR-924 was inversely correlated with that of DEFA5. Additionally, transfecting luciferase constructs containing the DEFA5 3′ UTR into Caco-2 cells resulted in decreased relative luciferase activity compared to transfecting empty vectors. Furthermore, creating a mismatch in the seed region of the miR-124 and miR-924 in the 3′UTR of DEFA5 resulted in restoration of luciferase activity. Finally, transfection of the miR-124 and miR-924 mimics significantly decreased DEFA5 mRNA expression and protein expression.

MicroRNAs regulate many processes, including differentiation and activation of the cells of the immune system. They have been demonstrated to play a role in a number of autoimmune diseases, including systemic lupus erythematosus, rheumatoid arthritis, and IBD [31-34]. Overall, the role of miRNAs as key negative regulators of inflammation, innate immunity, and epithelial function is being increasingly recognized [35]. A number of changes in miRNA expression in IBD and inflammation have been described [16-18]. As a result, miRNA regulation as an important component of intestinal epithelial innate immunity in epithelial cells is logical [36,37]. In a murine model, miR-146a regulated gut inflammation via the NOD2-sonic hedgehog (SHH) signaling pathway by suppressing SHH and ultimately resulting in increased pro-inflammatory cytokine expression [38]. Conversely, in HT29 cells, miR-122 decreased pro-inflammatory cytokines by downregulating LPS-induced NOD2 expression [39].

Specifically, we demonstrated that miRs-124 and -924 influence DEFA5 gene expression. Our data identifies an additional role for miR-124, which previously had been shown to be involved in IBD by regulation of STAT3 and regulation of CNS macrophages [40,41]. In children with UC and mice with experimental colitis, miR-124 levels were significantly decreased while STAT3 and downstream genes were up-regulated [40]. The IL6/STAT3 pathway activation has been shown to play a role in colitis by promoting inappropriate survival of T cells [42]. In a study examining CNS inflammation in vivo and in an experimental model, miR-124 was shown to play a central role in regulating microglial and macrophage quiescence [41]. miR-124 inhibited macrophage activation by binding the C/EBPα transcription factor [41]. This study defines a novel role for miR-924, which has not been previously described to regulate any genes.

A growing body of research demonstrates that defensins play a central role in IBD pathogenesis, in part related to changes in Wnt-signaling and mutations in NOD2 [43-45]. However, these mutations do not explain changes in defensin expression in a large proportion of patients, and these changes in defensin expression could perhaps be accounted for by dysregulation of miRNA regulation, as demonstrated in this study.

These changes in miRNA expression may explain alterations in α-defensin expression in IBD. Crohn's disease of the terminal ileum is unique because of the large number of Paneth cells and the highest density of microbes in the distal small intestine, which is otherwise low in the normal proximal small intestine [46,47].

In the ileal mucosa of CD, a decrease in antimicrobial activity could be attributed to decreased Paneth cell α-defensin expression [13]. The decreased levels of expression have been suggested to be predisposing factor for development of CD [13]. Additionally, these decreased levels in terminal ileum of CD patients with ileal disease involvement were noted to occur regardless of the presence of inflammation [14,43,48]. Levels of other Paneth cell products were unchanged or increased, indicating that the decrease in DEFA5 could be due to a defect in Paneth cell α-defensin regulation [14]. Alterations in miRNA regulation in IBD may explain defective α-defensin regulation in patients' whose disease cannot be attributed to mutations in NOD2.

One limitation of this study was the absence of an ideal experimental model. Colonic epithelial cell lines express α-defensin at relatively low levels. Because human Paneth cells do not survive under in vitro culture conditions, Caco-2 intestinal epithelial cells were selected since they share characteristics with small intestinal epithelial differentiation in vitro and constitutively express NOD2, much like Paneth cells [5,49]. Caco-2 cells also express fibroblast growth factor receptor-3, which is a critical regulator of Paneth cell differentiation during gut development [22,50]. Additionally, Caco-2 cells treated with FGF-2 provides the most suitable in vitro model available, as demonstrated in previous studies [22-24]. While the use of Caco-2 cells confirmed the regulation of DEFA5 by miRNAs, it may not exactly reflect small intestine physiology.

Overall, this study demonstrates that miRNAs are an important negative regulator of DEFA5. Our data establishes miRNA regulation of defensins and raises the possibility of dysfunctional miRNA regulation contributing to reduced DEFA5 levels seen in CD. Further examination of miRNA regulation of defensins, especially in inflammatory states, may contribute to a better understanding of the pathogenesis and may lead to the development of new diagnostic and therapeutic strategies for IBD patients.
